# No association of age-related maculopathy susceptibility protein 2/HtrA serine peptidase 1 or complement factor H polymorphisms with early age-related maculopathy in a Chinese cohort

**Published:** 2013-05-01

**Authors:** Jian-Huan Chen, Yunli Yang, Yuqian Zheng, Minghui Qiu, Mingliang Xie, Wenjie Lin, Mingzhi Zhang, Chi Pui Pang, Haoyu Chen

**Affiliations:** 1 Joint Shantou International Eye Center, Shantou University & the Chinese University of Hong Kong, Shantou, China; 2 Department of Ophthalmology and Visual Sciences, the Chinese University of Hong Kong, Hong Kong, China; 3 Nan’Ao People’s Hospital, Shantou, China

## Abstract

**Purpose:**

Single nucleotide polymorphisms (SNPs) of age-related maculopathy susceptibility protein 2/HtrA serine peptidase 1 (*ARMS2*/*HTRA1*) and complement factor H (*CFH*) have been reported to be associated with age-related macular degeneration (AMD). The purpose of this study was to investigate the association of *ARMS2/HTRA1* and *CFH* SNPs with early age-related maculopathy (ARM) in a Han Chinese cohort.

**Methods:**

The cohort consisted of 315 unrelated subjects, including 158 patients with early ARM and 157 recruited controls. Early ARM was diagnosed and graded according to the Age-Related Eye Disease Study criteria. Four SNPs in *ARMS2/HTRA1* and six SNPs in *CFH* previously reported to be associated with AMD were genotyped using TaqMan genotyping assays. Logistic regression implemented with the R statistical language was used for association analysis.

**Results:**

None of the *ARMS2/HTRA1* and *CFH* SNPs showed any significant association with early ARM (all p>0.453), with the odds ratios ranging from 0.88 to 1.17. None of the SNPs were associated with unilateral or bilateral early ARM or any grade of early ARM (all p>0.249).

**Conclusions:**

The association of *ARMS2/HTRA1* and *CFH* SNPs in early ARM was not detected in our cohort. The findings in the current study indicated that the effects of *ARMS2/HTRA1* and *CFH* in early ARM could be much lower compared to those in AMD.

## Introduction

Age-related maculopathy (ARM) is a common progressive disease that afflicts the elderly population worldwide. ARM has been divided into two stages, early and late [1]. Early stage ARM is characterized by drusen, the deposit of extracellular material between the retinal pigment epithelium and Bruch’s membrane, and hyperpigmentation or hypopigmentation of the retinal pigment epithelium (RPE). Late stage age-related maculopathy, also called age-related macular degeneration (AMD), is one of the most common causes of irreversible vision loss in developed countries [2]. AMD is further subdivided into dry and wet forms, which are characterized by geographic atrophy of the RPE and choroidal neovascularization, respectively.

By far, age is the most significant risk factor for ARM [3]. Cigarette smoking [4] and alcohol consumption [5-7] are two environmental risk factors identified by epidemiological studies. In the past decade, advances in genetic techniques have shown the contribution of genetic predisposition to the etiology of ARM. Genome-wide association studies have successfully identified susceptibility genes and loci for AMD [8]. The complement factor H gene (*CFH*) and 10q26 containing age-related maculopathy susceptibility protein 2 (*ARMS2*, also called *LOC387715*)/HtrA serine peptidase 1 (*HTRA1*) are two major loci associated with AMD identified by genome-wide association studies [9-13]. The associations have further been confirmed by numerous replication studies [8]. However, most of these studies focus on late stage ARM. The genetic susceptibility of early ARM remains less reported.

In the current study, we investigated the association of *ARMS2/HTRA1* and *CFH* polymorphisms, and environmental exposure including cigarette smoking and alcoholic consumption with prevalence of early ARM in a Chinese cohort. The possible association between the genetic factors and the course or severity of early ARM was also explored.

## Methods

### Patient recruitment and clinical examinations

This study was approved by the Ethics Committee of Joint Shantou International Eye Center and was conducted in accordance with the Declaration of Helsinki. Written consent was obtained from each participating subject after the nature of the study was explained.

The ARM patients included 39 male and 119 female with age between 50 and 80 years, and controls included 51 male and 106 female with age between 50 and 86 years (Table 1). The 315 study subjects were unrelated and included 158 patients with early ARM and 157 controls recruited at a satellite clinic at Nan’ao Island in Shantou, China. All participants underwent comprehensive ophthalmic examinations including best-corrected visual acuity, non-contact tonometry, slit-lamp biomicroscopy of anterior segment, and retina with mydriasis. Stereoscopic color fundus photographs were taken with a Topcon TRC-50EX retina camera (Topcon, Tokyo, Japan). ARM grading of the fundus photos followed the criteria of the Age-Related Eye Disease Study (AREDS) [14] by two independent masked trained graders (H.C. and M.Q.), and kappa statistics were calculated. Inconsistencies between the two graders were resolved by discussion. Subjects with maximal AREDS grades between 1 and 3 were diagnosed with early ARM. The controls had no drusen or pigment abnormality in either eye, and were aged more than 50 years old without family history of AMD. The patients with early ARM and the controls had no other eye diseases except mild senile cataract or refractive error between −6 and +6 diopter. Active cigarette smoking and alcohol consumption status in all participants was collected using a questionnaire. Active smoking was defined as smoking at least five cigarettes per day in the past one or more years [15]. Alcohol consumption history was defined as at least 12 alcoholic drinks in a year in a lifetime [7]. Peripheral blood was collected from all participants. Genomic DNA was extracted with the Qiamp Blood Kit (Qiagen, Hilden, Germany).

**Table 1:** Demographic and clinical features of the study subjects

Group	**N**	**Male/ Female**	**Age at diagnosis (year)**			**Cigarette**	**Alcoholic**	**Unilateral/**	**AREDS Grade**
			**range**	**median**	**mean ± SD**	**smoking (Yes/No)**	**drinking (Yes/No)**	**bilateral**	1/2/3
Control	157	51/106	50–86	60	61.7±9.2	40/114	37/120	-	-
ARM	158	39/119	50–80	60	61.6±8.2	31/123	24/133	47/111	73/72/13

ARM: age-related maculopathy; AREDS: Age Related Eye Diseases Study; SD: standard deviation

### Single nucleotide polymorphism genotyping

A total of ten single nucleotide polymorphisms (SNPs) previously reported to be associated with AMD [16-18], including rs2736911, rs10490924, rs11200638, and rs2672598 in *ARMS2/HTRA1* locus and rs3753394, rs800292, rs1061170, rs2274700, rs3753396, and rs1065489 in *CFH*, were genotyped by using the TaqMan SNP Genotyping assay (Applied Biosystems, Inc. [ABI], Foster City, CA) following the protocol suggested by the manufacturer. The quality control of the TaqMan assay reagents was performed by ABI before purchase. A subset of samples was selected to repeat the same TaqMan assays and further validated by using Sanger sequencing, to ensure high-quality genotyping.

### Statistical analysis

A Hardy–Weinberg test of each SNP and linkage disequilibrium analysis was conducted by using Haploview version 4.2 [19]. Haplotype block was defined using criteria proposed by Gabriel et al. [20]. Logistic regression controlled for age and sex implemented by the R statistical language version 2.15.1 was used to analyze the association of genetic polymorphisms and environmental exposure [21-23]. P values and odds ratios (ORs) were calculated using the additive model as previously described [23,24]. For p values less than 0.05, 10,000 permutations were used to correct multiple comparisons. A post-hoc power calculation was performed using GPower3 software.

## Results

### Demographic and clinical characteristics of study subjects

A total of 315 unrelated study subjects including 158 patients with early ARM and 157 controls were recruited. The demographic characteristics are shown in Table 1. There was no statistically significant difference in age and gender between the patients and the controls (all p>0.05, chi-square test and independent Student *t* test). After adjustments for sex and age, neither cigarette smoking nor alcohol consumption showed significant effects on disease onset of early ARM (OR=0.65, p=0.194 and OR=0.95, p=0.916, respectively). Among the 158 patients with early ARM, 47 had unilateral involvement and 111 bilateral. There were 73, 72, and 13 cases of AREDS grades 1, 2, and 3, respectively. The kappa of inter-grader agreement was 0.765.

### Hardy–Weinberg equilibrium and linkage equilibrium

**Figure 1:**
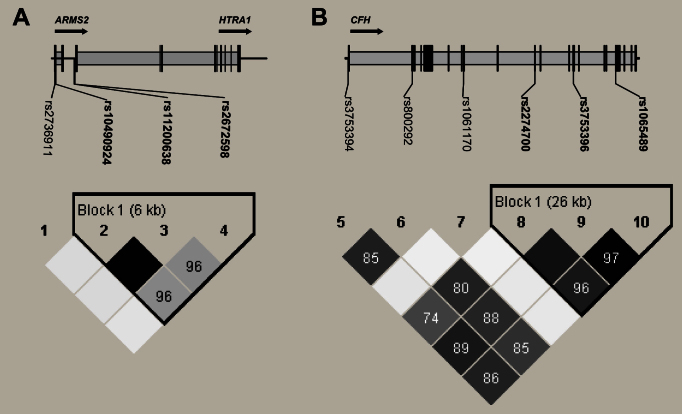
Linkage disequilibrium of age-related maculopathy susceptibility protein 2/HtrA serine peptidase 1 and complement factor H single nucleotide polymorphisms. **A**: The single nucleotide polymorphisms (SNPs) rs10490924, rs11200638, and rs2672598 in age-related maculopathy susceptibility protein 2/HtrA serine peptidase 1 (*ARMS2/HTRA1*) form a haplotype block in a 6 kb genomic region. (**B**) The SNPs rs10490924, rs11200638, and rs2672598 in complement factor H (*CFH*) form a long haplotype block in a 26 kb genomic region according to the criteria of the confidence intervals, an algorithm proposed by Gabriel et al. [20]. The solid line with an arrow indicates the transcription direction.

None of the ten SNPs in the current study showed deviation from Hardy–Weinberg equilibrium in the controls (p>0.05). Linkage disequilibrium analysis showed that the SNPs rs10490924, rs11200638, and rs2672598 in *ARMS2/HTRA1* form a haplotype block in a 6 kb genomic region (D′>0.96, Figure 1A). The SNPs rs2274700, rs3753396, and rs1065489 in *CFH* form a long haplotype block in a 26 kb genomic region (Figure 1B).

### Association with disease onset of early age-related maculopathy

The SNPs in *ARMS2/HTRA1* and *CFH* showed no significant association with early ARM (all p values>0.05, Table 2 and Table 3). The ORs of these SNPs ranged from 0.88 and 1.17 and were close to 1.

**Table 2:** Association of *ARMS2/HTRA1* SNPs with early ARM

SNP	Genotype	Frequency				Association	
		Control		ARM		OR	*P*
		N	%	N	%	(95% CI)	
rs2736911	CC	125	79.6	131	82.9	0.90	0.773
	CT	30	19.1	23	14.6	(0.53 - 1.5)	
	TT	2	1.3	4	2.5		
	C	280	89.2	285	90.2		
	T	34	10.8	31	9.8		
							
rs10490924	GG	60	38.2	60	38	1.12	0.553
	GT	78	49.7	71	44.9	(0.81 - 1.54)	
	TT	19	12.1	27	17.1		
	G	198	63.1	191	60.4		
	T	116	36.9	125	39.6		
							
rs11200638	GG	59	37.6	56	35.4	1.14	0.453
	AG	77	49	74	46.8	(0.83 - 1.58)	
	AA	21	13.4	28	17.7		
	G	195	62.1	186	58.9		
	A	119	37.9	130	41.1		
							
rs2672598	TT	72	45.9	81	51.3	0.88	0.511
	CT	69	43.9	61	38.6	(0.63 - 1.23)	
	CC	16	10.2	16	10.1		
	T	213	67.8	223	70.6		
	C	101	32.2	93	29.4		

The ORs of the minor alleles were calculated using the additive model controlling both sex and age. SNP: single nucleotide polymorphism; ARM: age-related maculopathy; OR: odds ratio; CI: confidence interval.

**Table 3:** Association of *Complement factor H* SNPs with early ARM

SNP	Genotype	Frequency		Association			
		Control		ARM		OR	*P*
		N	%	N	%	(95% CI)	
rs3753394	TT	49	31.2	51	32.3	1.04	0.868
	TC	74	47.1	68	43	0.76 - 1.42	
	CC	34	21.7	39	24.7		
	T	172	54.8	170	53.8		
	C	142	45.2	146	46.2		
							
rs800292	GG	51	32.5	55	34.8	1.08	0.680
	GA	79	50.3	66	41.8	0.79 - 1.48	
	AA	27	17.2	37	23.4		
	G	181	57.6	176	55.7		
	A	133	42.4	140	44.3		
							
rs1061170	CC	144	91.7	146	92.4	0.91	0.987
	CT	13	8.3	12	7.6	0.4 - 2.07	
	TT	0	0	0	0		
	C	301	95.9	304	96.2		
	T	13	4.1	12	3.8		
							
rs2274700	CC	49	31.2	48	30.4	1.17	0.372
	CT	84	53.5	75	47.5	0.85 - 1.6	
	TT	24	15.3	35	22.2		
	C	182	58	171	54.1		
	T	132	42	145	45.9		
							
rs3753396	GG	39	24.8	42	26.6	1.09	0.632
	AG	83	52.9	71	44.9	0.8 - 1.49	
	AA	35	22.3	45	28.5		
	G	161	51.3	155	49.1		
	A	153	48.7	161	50.9		
							
rs1065489	TT	39	24.8	41	25.9	1.08	0.690
	GT	82	52.2	73	46.2	0.79 - 1.48	
	GG	36	22.9	44	27.8		
	T	160	51	155	49.1		
	G	154	49	161	50.9		

The ORs of the minor alleles were calculated using the additive model controlling both sex and age. SNP: single nucleotide polymorphism; ARM: age-related maculopathy; OR: odds ratio; CI: confidence interval.

### Association with unilateral and bilateral early age-related maculopathy

The association of SNPs in *ARMS2/HTRA1* and *CFH* were further tested in patients with unilateral and bilateral ARM. No significance was observed in the *ARMS2/HTRA1* and *CFH* SNPs in the patients with unilateral ARM (p>0.622 and p>0.249, respectively, Table 4 and Table 5). Likewise, none of the *ARMS2/HTRA1* and *CFH* SNPs showed significance in the patients with bilateral ARM (p>0.709 and p>0.348, respectively, Table 4 and Table 5)

**Table 4:** Association of *ARMS2/HTRA1* SNPs with unilateral and bilateral early ARM

Genotype frequency	Genotype frequency						Association			
	CON		Unilateral ARM		Bilateral ARM		Unilateral ARM		Bilateral ARM	
	N	%	N	%	N	%	OR(95% CI)	*P*	OR(95% CI)	*P*
rs2736911										
CC	125	79.6	39	83	92	82.9	0.78	0.538	0.94	0.809
CT	30	19.1	8	17	15	13.5	(0.32 - 1.67)		(0.54 - 1.59)	
TT	2	1.3	0	0	4	3.6				
										
rs10490924										
GG	60	38.2	21	44.7	39	35.1	0.98	0.931	1.22	0.277
GT	78	49.7	18	38.3	53	47.7	(0.6 - 1.58)		(0.85 - 1.76)	
TT	19	12.1	8	17	19	17.1				
										
rs11200638										
GG	59	37.6	20	42.6	36	32.4	1.03	0.898	1.24	0.249
AG	77	49	18	38.3	56	50.5	(0.64 - 1.65)		(0.86 - 1.78)	
AA	21	13.4	9	19.1	19	17.1				
										
rs2672598										
TT	72	45.9	23	48.9	58	52.3	0.88	0.622	0.86	0.429
CT	69	43.9	20	42.6	41	36.9	(0.52 - 1.45)		(0.59 - 1.24)	
CC	16	10.2	4	8.5	12	10.8				

The ORs of the minor alleles were calculated using the additive model controlling both sex and age. SNP: single nucleotide polymorphism; ARM: age-related maculopathy; OR: odds ratio; CI: confidence interval.

**Table 5:** Association of *Complement facor H* SNPs with unilateral and bilateral early ARM

Genotype frequency			Genotype frequency				Association			
	CON		Unilateral ARM		Bilateral ARM		Unilateral ARM		Bilateral ARM	
	N	%	N	%	N	%	OR(95% CI)	*P*	OR(95% CI)	*P*
rs3753394										
TT	49	31.2	17	36.2	34	30.6	0.95	0.835	1.07	0.712
TC	74	47.1	19	40.4	49	44.1	(0.6 - 1.5)		(0.76 - 1.49)	
CC	34	21.7	11	23.4	28	25.2				
										
rs800292										
GG	51	32.5	17	36.2	38	34.2	1.01	0.966	1.07	0.684
GA	79	50.3	20	42.6	46	41.4	(0.63 - 1.61)		(0.76 - 1.51)	
AA	27	17.2	10	21.3	27	24.3				
										
rs1061170										
CC	144	91.7	44	93.6	102	91.9	0.78	0.709	0.99	0.989
CT	13	8.3	3	6.4	9	8.1	(0.17 - 2.57)		(0.39 - 2.41)	
TT	0	0	0	0	0	0				
										
rs2274700										
CC	49	31.2	16	34	32	28.8	1.09	0.721	1.19	0.348
CT	84	53.5	21	44.7	54	48.6	(0.67 - 1.78)		(0.83 - 1.7)	
TT	24	15.3	10	21.3	25	22.5				
										
rs3753396										
GG	39	24.8	14	29.8	28	25.2	1.07	0.777	1.09	0.632
AG	83	52.9	19	40.4	52	46.8	(0.67 - 1.71)		(0.77 - 1.55)	
AA	35	22.3	14	29.8	31	27.9				
										
rs1065489										
TT	39	24.8	14	29.8	27	24.3	1.06	0.817	1.09	0.644
GT	82	52.2	19	40.4	54	48.6	(0.66 - 1.68)		(0.77 - 1.54)	
GG	36	22.9	14	29.8	30	27				

The ORs of the minor alleles were calculated using the additive model controlling both sex and age. SNP: single nucleotide polymorphism; ARM: age-related maculopathy; OR: odds ratio; CI: confidence interval.

### Association with Age-Related Eye Disease Study grading of early age-related maculopathy

No significant association of the *ARMS2*/*HTRA1* and *CFH* SNPs with any grade of ARM was found in the current cohort (all p>0.125, Table 6 and Table 7).

**Table 6:** Association of *ARMS2/HTRA1* SNPs with AREDS grading of early ARM

SNP	Genotype frequency									Association					
	Genotype	CON		ARM Grade 1		ARM Grade 2		ARM Grade 3		ARM Grade 1		ARM Grade 2		ARM Grade 3	
		N	%	N	%	N	%	N	%	OR (95%CI)	*P*	OR (95%CI)	*P*	OR (95%CI)	*P*
rs2736911	CC	125	79.6	60	82.2	60	83.3	11	84.6	0.94	0.858	0.89	0.727	0.70	0.641
	CT	30	19.1	11	15.1	10	13.9	2	15.4	(0.49 - 1.74)		(0.46 - 1.66)		(0.11 - 2.51	
	TT	2	1.3	2	2.7	2	2.8	0	0						
															
rs10490924	GG	60	38.2	29	39.7	26	36.1	5	38.5	1.1	0.645	1.17	0.466	1.11	0.819
	GT	78	49.7	31	42.5	34	47.2	6	46.2	(0.73 - 1.67)		(0.77 - 1.77)		(0.46 - 2.6	
	TT	19	12.1	13	17.8	12	16.7	2	15.4						
															
rs11200638	GG	59	37.6	27	37	24	33.3	5	38.5	1.14	0.539	1.19	0.414	1.07	0.877
	AG	77	49	32	43.8	36	50	6	46.2	(0.75 - 1.71)		(0.78 - 1.8)		(0.44 - 2.49)	
	AA	21	13.4	14	19.2	12	16.7	2	15.4						
															
rs2672598	TT	72	45.9	37	50.7	39	54.2	5	38.5	0.93	0.751	0.75	0.205	1.27	0.565
	CT	69	43.9	27	37	28	38.9	6	46.2	(0.61 - 1.42)		(0.48 - 1.16)		(0.54 - 2.89)	
	CC	16	10.2	9	12.3	5	6.9	2	15.4						

The ORs of the minor alleles were calculated using the additive model controlling both sex and age. SNP: single nucleotide polymorphism; ARM: age-related maculopathy; AREDS: Age related eye diseases study; OR: odds ratio; CI: confidence interval.

**Table 7:** Association of *Complement factor H* SNPs with AREDS grading of early ARM

SNP	Genotype Frequency									Association					
	Genotype	CON		ARM Grade 1		ARM Grade 2		ARM Grade 3		ARM Grade 1		ARM Grade 2		ARM Grade 3	
		N	%	N	%	N	%	N	%	OR (95%CI)	*P*	OR (95%CI)	*P*	OR (95%CI)	*P*
rs3753394	TT	49	31.2	20	27.4	26	36.1	5	38.5	1.22	0.312	0.85	0.415	1.07	0.863
	TC	74	47.1	31	42.5	33	45.8	4	30.8	(0.83 - 1.8)		(0.57 - 1.25)		(0.48 - 2.35)	
	CC	34	21.7	22	30.1	13	18.1	4	30.8						
															
rs800292	GG	51	32.5	21	28.8	28	38.9	6	46.2	1.22	0.328	0.91	0.641	1.02	0.963
	GA	79	50.3	33	45.2	30	41.7	3	23.1	(0.82 - 1.83)		(0.61 - 1.35)		(0.44 - 2.29)	
	AA	27	17.2	19	26	14	19.4	4	30.8						
															
rs1061170	CC	144	91.7	65	89	69	95.8	12	92.3	1.43	0.453	0.5	0.29	0.96	0.969
	CT	13	8.3	8	11	3	4.2	1	7.7	(0.54 - 3.62)		(0.11 - 1.61)		(0.05 - 5.58)	
	TT	0	0	0	0	0	0	0	0						
															
rs2274700	CC	49	31.2	18	24.7	25	34.7	5	38.5	1.39	0.125	1.01	0.958	0.9	0.807
	CT	84	53.5	36	49.3	33	45.8	6	46.2	(0.92 - 2.11)		(0.67 - 1.53)		(0.36 - 2.12)	
	TT	24	15.3	19	26	14	19.4	2	15.4						
															
rs3753396	GG	39	24.8	15	20.5	22	30.6	5	38.5	1.37	0.125	0.89	0.556	0.92	0.84
	AG	83	52.9	33	45.2	34	47.2	4	30.8	(0.92 - 2.08)		(0.59 - 1.32)		(0.4 - 2.09)	
	AA	35	22.3	25	34.2	16	22.2	4	30.8						
															
rs1065489	TT	39	24.8	15	20.5	21	29.2	5	38.5	1.37	0.129	0.9	0.624	0.76	0.524
	GT	82	52.2	33	45.2	35	48.6	5	38.5	(0.92 - 2.06)		(0.6 - 1.35)		(0.32 - 1.74)	
	GG	36	22.9	25	34.2	16	22.2	3	23.1						

The ORs of the minor alleles were calculated using the additive model controlling both sex and age. SNP: single nucleotide polymorphism; ARM: age-related maculopathy; AREDS: Age related eye diseases study; OR: odds ratio; CI: confidence interval.

## Discussion

Our results showed no association between ten SNPs in the two major AMD-associated genetic loci, *CFH* and *ARMS2*/*HTRA1,* with either disease onset or course of early ARM in a Han Chinese cohort. The association of *CFH* SNP rs1061170 (Y402H) with late stage AMD was first discovered with a genome-wide association study. Later, it was reported that the Y402H variation was also associated with soft drusen in Caucasians [25-27]. However, the association of Y402H with hard drusen remains controversial. Positive [25] and negative [27,28] results have been reported in investigating the association of Y402H and hard drusen in Caucasians. There was ethnic variation in the minor allele frequency of Y402H and its association with AMD. In a Latino population, Y402H was reported to be associated only with bilateral early AMD but not unilateral early AMD [29]. The minor allele frequency of Y402H is low in East Asians and contributes to a small portion of AMD. In a multiethnic study in the United States, Y402H was associated with early AMD in Caucasians and Hispanics but not Chinese or Black groups [30]. In Chinese, the results are controversial. In a report of Taiwanese Chinese, Y402H was associated with early AMD [31]. In the Beijing Eye Study, Y402H was associated with bilateral but not unilateral soft drusen [32]. In another report, no SNP in *CFH* was associated with drusen [33]. Similar results were observed at the genotype level. The absence of a homozygous minor genotype of rs1061170 and the high frequency of the homozygous major genotype in controls were similar to those reported in other East Asian populations, including Korean, Japanese, and other Chinese cohorts. In early ARM, the frequency of the homozygous minor genotype of rs1061170 was reported to be 6% in the Taiwan study [31]; however, the genotype was shown to be rare in rare (1.4%) in the Beijing Eye study [32]. Our study also found no association of Y402H in *CFH* with early AMD, either unilateral or bilateral. It was reported that in Chinese, the significant AMD-associated SNP was not Y402H but other SNPs, such as rs3753394, rs800292, and rs2274700 [34]. In our study, we also excluded the association of these SNPs with early ARM.

The 10q26 locus is another major locus associated with AMD. Two genes in this locus were associated with AMD, *ARMS2* [35] and *HTRA1* [12,13]. The rs10490924 at *ARMS2* and rs11200638 at *HTRA1* were in high linkage disequilibrium. Currently, there is still debate over which gene if not both contributes to AMD risk. It was reported that in a population in Utah the rs11200638 at *HTRA1* was associated with soft drusen [36]. In the AREDS cohort, rs10490924 at *ARMS2* was associated with large drusen only but not intermediate drusen [37]. However, in a Russian and Greek population, *ARMS2* was associated only with late AMD but not early AMD [38,39]. In a Chinese population, rs11200638 at *HTRA1* was not associated with drusen [40]. In a Danish population, the presence of 20 or more small hard drusen was not observed with rs10490924 at *ARMS2* or rs11200638 at *HTRA1* [28]. In this study of a Chinese cohort, we found that rs10490924, rs11200638, rs2672598, and rs2736911 were not associated with early AMD.

The ten SNPs in *CFH* and *ARMS2*/*HTRA1* have been previously reported to be significantly associated with AMD [9-13,17]. In our cohort of early ARM, no association of the ten SNPs in *ARMS2*/*HTRA1* and *CFH* was detected. The ORs of association with ARM was close to 1, probably suggesting that the effect sizes of *CFH* and *ARMS2/HTRA1* SNPs in early ARM could be much lower compared to those previously reported for AMD in Chinese [16-18,31]. Furthermore, our findings indicated that *ARMS2/HTRA1* and *CFH* SNPs were unlikely to be involved in disease severity or course. The ORs of the SNPs rs10490924 and rs11200638 increased in bilateral early ARM compared to unilateral ARM. However, the association did not reach statistical significance, and the effect sizes were similarly much smaller compared to those in AMD. The ORs did not show a consistent trend with the grade or increased early ARM, which thus further suggested the lack of involvement of the ARMS2*/HTRA1* and *CFH* SNPs in disease severity. Lower post-hoc power was observed in the unilateral early ARM test, probably due to the small sample size. Although rs2736911 in *ARMS2* and rs1065489 in *CFH* had relatively lower power (56% and 66%, respectively), most of the SNPs in the current study showed high post-hoc power (≥73%) in the total early ARM test and the bilateral early ARM test (Appendix 1 and Appendix 2).

Ethnic variation in prevalence has been revealed by the epidemiology of ARM. The Beijing Eye Study reported the prevalence of early ARM in Chinese individuals aged 40 or more years reported as 1.4% [41]. Similarly, early ARM has a lower prevalence in other East Asians such as Japanese compared to Caucasians of European descent [42]. Such variation is in line with the difference in genetics of early ARM found in the current study.

In addition to genetic factors, environmental exposure such as cigarette smoking has been shown to be involved in the development of AMD [17,43-45]. However, active cigarette smoking did not show significant effects on disease onset of early ARM in the current study (with an OR close to 1). Alcohol consumption showed an OR equal to 0.65, with the p value equal to 0.194. Further investigation is needed to study the effects of alcohol consumption in early ARM.

In conclusion, our results showed no association between the two major AMD associated loci and early ARM in terms of disease onset or course. In contrast to the strong genetic contribution to AMD, these findings suggest a much smaller effect of the two major AMD-associated loci on the etiology of early ARM, and thus implicate differential pathophysiological mechanisms between early ARM and AMD.

## Appendix 1. Post Hoc power calculation for *HTRA1/AMS2* SNPs in the current study.

*Effect size from Yang Z. et al. was used for Post Hoc power calculation. **Effect size from Tam P.O.S. et al. was used for Post Hoc power calculation. To access the data, click or select the words “Appendix 1.”

## Appendix 2. Post Hoc power calculation for *CFH* SNPs in the current study.

*Effect size from Ng T.K. et al. was used for Post Hoc power calculation. **Effect size from Lin J.M.. et al. was used for Post Hoc power calculation. To access the data, click or select the words “Appendix 2.”
